# Perceived stress and affective experience in Italian teachers during the COVID-19 pandemic: correlation with coping and emotion regulation strategies

**DOI:** 10.1007/s10212-022-00661-6

**Published:** 2022-12-03

**Authors:** Linda Messineo, Crispino Tosto

**Affiliations:** Istituto per le Tecnologie Didattiche, Consiglio Nazionale delle Ricerche, Via Ugo La Malfa, 153, 90146 Palermo, Italy

**Keywords:** Coping strategies, COVID-19, Emotion regulation, Perceived stress, Positive and negative affect, Teachers

## Abstract

The COVID-19 pandemic has represented a source of stress for teachers by adding new challenges. The objective of this study was to assess the association between emotion regulation and coping strategies, on the one hand, and perceived stress and affective experience on the other among teachers during the COVID-19 pandemic. A sample of 1178 of Italian pre-primary, primary, and secondary school teachers completed an online survey. Three hierarchical linear regression analyses were run to evaluate teachers’ emotion regulation, coping strategies, years of teaching experience, perceived workload, and perceptions about online teaching in predicting their perceived stress and positive and negative affect. The findings showed that cognitive reappraisal and positive attitude were associated with a lower level of perceived stress and negative affect and a higher level of positive affect. Problem orientation strategies were also associated with a higher level of positive affect. By contrast, expressive suppression was correlated with a higher level of perceived stress. Avoidance coping strategies were associated with higher perceived stress and negative affect and a lower level of positive affect. The perceived burden of online teaching was positively related with perceived stress and negative affect. Confidence in using educational technologies predicted lower levels of perceived stress, and previous online teaching experience was positively correlated with positive affect. The findings of this study could be useful for implementing teacher training programmes on emotion regulation and coping strategies to reduce stress and promote positive affect.

## Introduction

The COVID-19 pandemic has had a severe impact on the educational system, in which different changes in educational practices and routines have been needed (OECD, 2021). Teaching is recognized as a highly stressful profession (Cooper & Travers, 2012). The pandemic has added a number of complex and unexpected challenges that have had an impact on the psychological state of teachers (Jakubowski & Sitko-Dominik, 2021; Kim et al., 2022). In this context, teachers have had to adopt new strategies and approaches for the management of unpredictable and complex work-related situations. From the initial emergency period of the pandemic, teachers adopted specific preventive actions to prevent infections. The schools were closed in some periods, and teachers were driven to quickly adapt to teaching online. Remote work significantly increased teachers’ workload. Indeed, teachers were faced with the need to modify their teaching methods and educational tools without adequate specific training (Espino-Díaz et al., 2020). All these situations represented a source of stress for teachers (MacIntyre et al., 2020). The negative impact of the COVID-19 pandemic on the wellbeing of teachers can represent a risk factor not just for teachers, but also for the entire education system. The present study aimed to measure teachers’ perceived stress and affective experience, and assessed emotional regulation and coping strategies as possible predictive factors during the COVID-19 pandemic. The study also sought to examine the value of their experience of online teaching and perceived workload in predicting both teachers’ stress and affective experience.

## Theoretical background

### Emotional experience and emotion regulation

Highly stressful events, such as the COVID-19 pandemic, elicit emotional states (Lazarus, 1999). During the last few years, it has been observed that positive emotions co-occur with negative emotions during stressful periods (Folkman, 2008). Emotions are multi-componential processes. Emotions are responses to salient events and situations and are characterized by subjective experiences and biological reactions. Teachers regularly experience both positive and negative emotions (Frenzel, 2014). Recent research has shown that teachers’ positive emotions are associated with their self-efficacy, job satisfaction, work engagement, enthusiasm, and wellbeing; conversely, negative emotions are related to lower self-efficacy and lower work engagement (Burić & Macuka, 2018; Burić & Moè, 2020). The capability to regulate emotions represents a crucial aspect for teachers to effectively cope with stress (Sutton & Harper, 2009). Emotion regulation can be defined as the capability to evaluate and modulate emotional responses such as attenuating, intensifying, or maintaining positive and negative emotions in response to significant stimuli from the environment (Gross, 2014). Specifically, emotion regulation refers to the processes that influence how people experience and express positive and negative emotions (Gross, 1998; 2014). According to the process model of emotion regulation worked out by Gross, there are different typologies of emotional regulation strategies that can be distinguished, based on the time of their activation along the time span of the emotional experience, as antecedent-focused regulations and response-focused regulations (Gross, 1998). Each emotion starts with the evaluation of a stimulus that activates subjective, behavioural, and physiological responses. Emotional regulation strategies are behavioural and cognitive processes that make it possible to regulate the intensity, duration, and expression of emotions, which is useful for individual wellbeing and mental health (Gross, 2014)*.* Antecedent-focused regulation occurs early, before the emotional response has been entirely generated, and focuses on modifying the effects of emotional cues. These strategies make it possible to regulate emotional responses before the bio-physiological components are activated and behavioural responses are expressed. There are different types of antecedent-focused regulations. In this study, we refer to cognitive reappraisal, which consists in re-evaluating the meaning of a specific situation that elicits an emotion (Gross & John, 2003). Response-focused strategies occur after the emotion is already in progress and emotional responses have been activated. Expressive suppression is a response-focused strategy and consists in inhibiting expressive behaviours, such as facial or verbal expressions (Gross, 1998). Unlike cognitive reappraisal, suppression should not decrease emotional experience. Research has shown that expressive suppression is likely to be less effective in modifying negative emotions than cognitive reappraisal (Hu et al., 2014). Expressive suppression is also expected to be associated with poorer perceived wellbeing, lower interpersonal functioning, and greater levels of negative affect (Gross, 2015; Gross & John, 2003; Hu et al., 2014). By contrast, research has shown that cognitive reappraisal is associated with greater experience of positive affect and lower negative affect (Gross & John, 2003), and perceived high wellbeing and social support (Gross 2014; Hu et al., 2014).

Teachers feel a variety of positive and negative emotions during their everyday work in the learning environment and they use a variety of emotion regulation strategies to manage and regulate their emotions (Frenzel, 2014; Sutton & Wheatley, 2003). Some studies have investigated the strategies that teachers use to regulate their emotions. For example, Sutton et al. (2009) reported a series of studies, and they observed that teachers with different levels of experiences and from different levels of schools (early childhood, middle, and high) use both antecedent-focused and response-focused strategies in teaching. Chang and Taxer (2020) examined how teachers from elementary, middle, and secondary schools in the USA regulate their emotions in response to student misbehaviour. They observed that teachers who stated they usually use a high level of reappraisal and low levels of suppression report the lowest level of negative affective experiences, such as anger or emotional exhaustion, in the context of student misbehaviour. Other studies have investigated the impact of emotion regulation on teachers’ wellbeing. For example, in a study conducted with Spanish teachers from several grades, it was found that teachers with higher emotion regulation ability, defined as the capability to perceive, understand, and regulate their own and others’ emotions, reported a lower level of psychological symptoms (Mérida-López et al., 2017). In another study, carried out with fulltime teachers from a Midwestern state in the USA, with a mean number of 5 years’ teaching, it was found that using cognitive reappraisals was negatively associated with teacher burnout (Chang, 2020).

### Teacher stress

Teaching is a profession with a high prevalence of work-related stress (Cooper & Travers, 2012). Stress is experienced when a person feels that the demand of a situation is perceived as excessive when compared to the available coping resources (Lazarus & Folkman, 1984). Stress and negative emotions in teachers can lead to personal consequences, such as professional burnout and decreased job satisfaction (Klassen & Chiu, 2010). In addition, teacher stress can affect students’ performance, health, and psychological wellbeing (Wentzel, 2010). Indeed, teachers play a crucial role not only in the learning process of the different disciplines but also in the cognitive, emotional, and psychological development of their students (O'Connor, 2008). Different studies have shown that teachers’ wellbeing is related to classroom effectiveness and students’ wellbeing. For example, a study found that improved teacher wellbeing is associated with better student wellbeing and fewer student psychological difficulties (Harding et al., 2019). Conversely, a study showed that teachers’ high stress and burnout and a lower level of reported coping skills are associated with the worst student outcomes (Herman et al., 2018). Moreover, a systematic review showed that teacher burnout is related not only to lower academic achievement, but also to lower quality motivation (Madigan & Kim, 2021).

The COVID-19 pandemic represents a source of stress for teachers. Research has focused on stress among teachers during the different phases of the pandemic. For example, a study performed during the first phase of the pandemic in China found that the prevalence of acute stress symptoms in primary and secondary school teachers was 9.1% (Zhou & Yao, 2020). In another study, the psychological risk of teachers in Spain and Mexico was analysed (Prado-Gascó et al., 2020); teachers reported having a work overload as an important source of psychological risk. After a long period, schools were periodically re-opened, and teachers conducted face-to-face classes. Some studies were carried out after the reopening of schools during COVID-19. In a study conducted by Ozamiz-Etxebarria et al. (2021), the symptomatology of teachers in the Basque Autonomous Community was assessed. The results showed that a high percentage of teachers suffered from stress, anxiety, and depression. In this study, the authors found an age-related difference in anxiety, with older teachers showing higher levels (of anxiety). By contrast, older and younger teachers showed a higher level of stress than middle-aged ones. Moreover, professional variables such as job stability, teaching sector, and the typology of school in which they teach also influenced their psychological state. In a study by Jakubowski and Sitko-Dominik (2021), carried out during the first and second waves of the COVID-19 pandemic in Poland, it was found that primary and secondary teachers experienced at least mild levels of stress, anxiety, and depression.

### Coping strategies

The COVID-19 pandemic is a source of stress with which people have to cope. Coping strategies are defined as cognitive and behavioural efforts to manage internal or external stressors that are appraised as exceeding the resources of a person (Lazarus & Folkman, 1984). Coping involves conscious, purposeful actions to reduce the perceived discrepancy between internal/external demands and resources (Lazarus, 1999). Coping strategies have been distinguished into different categories, such as problem-focused versus emotion-focused, positive versus negative, and approach versus avoidance (Skinner et al., 2003). Lazarus and Folkman (1984) distinguished problem-focused from emotion-focused coping strategies. In the first case, individuals directly manage the stressor; in the latter, they regulate the arising emotions as a consequence of the stressful encounter. Some authors investigating use of coping strategies distinguish between positive and negative coping strategies, emphasizing their relative effectiveness (Skinner et al., 2003). While positive coping strategies are a constructive, direct, and positive manner to deal with problems, negative coping strategies focus on the tendency to react in an avoidant or unconstructive manner to stressful events. Coping strategies are thus protective factors when they facilitate management of stressful events (Folkman & Moskowitz, 2004). Coping strategies such as having a positive attitude towards the stressful situation (Babore et al., 2020), applying active coping strategies, and seeking emotional social support (Park et al., 2020) are predictors of low levels of distress caused by COVID-19. A cross-sectional study among residents of Wuhan (China) showed that adoption of positive coping strategies can be influenced by the education level (Fu et al., 2020). In this study, use of positive coping strategies by people with a higher level of education seems to encourage adoption of more proactive coping models, such as reading, physical activity, and seeking psychological support from the family. Conversely, people with negative coping styles showed higher levels of psychological distress during the emergency (Wang et al., 2020). For example, high levels of psychological distress were identified in people who spent more than 6 h per day searching for information about COVID-19, receiving poor social support, and rarely using active coping strategies to face the difficulties positively (Yu et al., 2020). In the present study, we refer to the distinction between approach and avoidance coping strategies (Skinner et al., 2003); indeed, as highlighted in a review by Carton and Fruchart (2014), in studies carried out to investigate use of coping strategies in teachers, these strategies are generally differentiated into approach coping ones, such as problem solving, and avoidance coping ones, such as distancing. In a study carried out during the COVID-19 pandemic with a sample of language teachers (MacIntyre et al., 2020), the authors found that positive psychological outcomes, such as wellbeing and health, correlated positively with approach coping strategies and negatively with avoidance coping strategies. Moreover, negative psychological outcomes, such as stress and anxiety, were significantly correlated with avoidance coping strategies. In this study, the authors also observed that use of avoidance coping strategies increased as stress increased.

While emotion regulation and coping strategies are different constructs, there are some overlaps between them (Compas et al., 2014). In both of them, a regulatory process is a crucial concept, but it takes place in response to different situations. Emotion regulation occurs in response to stressful and non-stressful situations to evaluate and modulate both positive and negative emotions experienced by people. Coping strategies are activated in response to a stressor. Furthermore, coping is a broader construct that includes some types of emotion-focused strategies, such as avoidance and positive reappraisal. Hence, some strategies, such as avoidance and emotional suppression, are included in both emotion regulation and coping strategies. Consequently, research shows specific relationships between emotional regulation strategies and coping strategies that share conceptual overlap; for instance, cognitive reappraisal is positively correlated with positive reinterpretation and problem-focused coping strategies, and suppression is correlated negatively with venting coping strategy (Balzarotti et al., 2010; Gross & John, 2003).

## Objectives and hypotheses

This study mainly aimed at evaluating the associations between emotion regulation and coping strategies, on the one hand, and perceived stress and negative affect and positive affect, on the other, after controlling for socio-demographic variables, in a sample of Italian teachers during the COVID-19 pandemic. Additionally, the study assessed the associations of factors related to teachers’ experience of online teaching and perceived workload with perceived stress and affective experience. With regard to the predictive value of emotion regulation, we first hypothesized that use of cognitive reappraisal would be associated with a lower level of perceived stress, and positively correlated with positive affect and negatively with negative affect respectively. By contrast, we hypothesized that use of expressive suppression would be associated with a higher level of perceived stress, and negatively correlated with positive affect and positively with negative affect respectively. Regarding coping strategies, we hypothesized that use of avoidance-oriented strategies would be a predictive factor of higher levels of perceived stress and negative affect; we also hypothesized that use of active coping strategies, such as problem orientation and positive attitude, would be positively associated with positive affect and with a lower level of perceived stress. Finally, we expected factors related to online teaching, especially previous experience and perceived burden of online teaching, to be significantly associated with the selected outcomes.

## Method

### Design

The present study was a cross-sectional study conducted between March and May 2021. Teachers in service at Italian pre-primary, primary, and secondary schools were approached using a convenience sampling, supported by a snowball sampling technique. In Italy, pre-primary schools include education for children aged 3–5. Primary education runs from ages 6–10, and secondary education covers ages 11 through 18 and is divided into two levels, first and second grade. The link to the online survey to be completed for the purposes of the research was disseminated through the institutional website and personal networks of the research team. In addition, the link to the survey was sent by institutional email to around 30% of the schools available in each Italian region. In the email, the heads of the schools were invited to disseminate the survey among the teaching staff of their schools. On the first page of the survey, in addition to the objectives of the research, references to the current Italian and European privacy legislation were included. Teachers were asked to give voluntary and informed consent before answering the survey. Participants were guaranteed anonymity, and could abandon the research at any time, without any consequences. It took about 15 min to complete the whole survey.

### Ethics

The study was conducted in accordance with the Declaration of Helsinki and authorized by the Ethic Committee of Azienda Ospedaliera Universitaria Policlinico Paolo Giaccone of Palermo, Italy.

### Participants

A total of 1497 teachers at pre-primary, primary, and secondary schools answered the questionnaires. Responses from 309 participants were excluded from the analyses because they were incomplete; specifically, those participants who did not complete at least one of the questionnaires included in the survey were removed. Responses from 9 additional teachers were excluded as reporting unlikely values in the variables measuring participants’ age and years of teaching. One additional case indicated the category “other” to identify gender; this unique case was removed to adjust regression analyses for gender. Hence, analyses were run on a total of 1178 participants. Participants’ mean age was 47.31 years (*SD* = 10.02, range 20–67) and most of them were female (*n* = 988, 83.9%). Additional participants’ characteristics, also differentiated by gender, are shown in Table 1. Most of them, regardless of whether they were male or female, were married or living with a partner and reported having at least one child. On average, they had been teaching for 17 years and mainly at the primary school level or above.

**Table 1:** Participants’ characteristics

Variable	Gender											
	Female (*n* = 988)				Male (*n* = 190)				Total (*N* = 1178)			
	*n*	%	*M* (*SD*)	Range	*n*	%	*M* (*SD*)	Range	*n*	%	*M* (*SD*)	Range
Age			47.59 (9.96)	23–67			45.87 (10.26)	20–66			47.31 (10.02)	20–67
Marital status												
Married/living with a partner	715	72.37			115	60.53			830	70.46		
Single/never married	184	18.62			65	34.21			249	21.14		
Separated/divorced	75	7.59			9	4.74			84	7.13		
Widowed	14	1.42			1	0.53			15	1.27		
Children												
No	320	32.39			92	48.42			412	34.97		
< 18	296	29.96			26	13.68			322	27.33		
> 18	289	29.25			63	33.16			352	29.88		
Of all ages	83	8.40			9	4.74			92	7.81		
Location												
North	648	65.59			128	67.37			776	65.87		
Centre	188	19.03			41	21.58			229	19.44		
South and Islands	152	15.38			21	11.05			173	14.69		
Years of teaching			17.81 (11.72)	0–45			13.86 (10.59)	0–40			17.18 (11.63)	0–45
School level												
Pre-primary	119	12.04			3	1.58			122	10.36		
Primary	349	35.32			12	6.32			361	30.65		
Low secondary	211	21.36			45	23.68			256	21.73		
High secondary	309	31.28			130	68.42			439	37.27		
Type of contract												
Permanent contract	713	72.17			114	60.00			827	70.20		
Fixed-term contract	275	27.83			76	40.00			351	29.80		

*M*, mean; *SD*, standard deviation

### Measures

The teachers were asked to answer an anonymous online survey to detect socio-demographic factors, teaching career information, perceived workload, attitude towards online teaching, emotion regulation, positive and negative affect, perceived stress, and coping strategies.

#### Socio-demographic factors and teaching career information

A set of questions were used to assess teachers’ socio-demographic characteristics (gender, age, marital status, having children, geographical location) and collect information about their teaching career (school level, type of contract, and years of teaching).

#### Perceived workload and attitude towards online teaching

The following question, with the response options ranging from 1 (*much decreased*) to 7 (*much increased*), was administered to assess teachers’ perceived workload as a consequence of the COVID-19 pandemic: “Has the workload increased due to the COVID-19 emergency?” Six questions rated on an 11-point Likert-type scale ranging from 0 (*absolutely disagree*) to 10 (*absolutely agree*) were administered to assess participants’ perceptions and attitudes towards online teaching. These six questions were adapted, with the authors’ authorization, from some of the items included in a survey aimed to explore school and college teachers’ wellbeing and workload during the lockdown in England (See et al., 2020).

#### Emotion regulation

The Italian version of the Emotion Regulation Questionnaire (ERQ) was administered to measure the emotion regulation strategies used by teachers (Balzarotti et al., 2010; Gross & John, 2003). The ERQ consists of ten items, and it is divided into two scales assessing two emotion regulation strategies, specifically cognitive reappraisal and expressive suppression. The cognitive reappraisal scale comprises six items and the expressive suppression scale comprises 4 items. Teachers were asked to rate their agreement about each item on a 7-point Likert scale ranging from 1 (*strongly disagree*) to 7 (*strongly agree*). In the present study, Cronbach’s alphas for reappraisal and suppression scales were found to be equal to 0.84 and 0.72 respectively.

#### Affective experience

The Italian version of the Positive and Negative Affect Schedule (PANAS) was administered to measure teachers’ affective experience (Terracciano et al., 2003; Watson et al., 1988). Positive affect and negative affect are considered emotional components of subjective wellbeing. Positive affect and negative affect are two independent and uncorrelated factors of affective experience (Watson et al., 1988). Positive affect comprises emotional and mood states, like enthusiasm and joy. Negative affect includes aversive emotional states and moods, such as distress and nervousness. The scale is composed of twenty items, with ten items assessing positive affect (e.g., enthusiastic, determined) and ten items assessing negative affect (e.g., irritable, distressed). Teachers were asked to rate each item on a 5-point Likert scale ranging from 1 (*very slightly or not at all*) to 5 (*extremely*) to assess the extent to which each affect has been felt during the past month. Cronbach’s alphas for both the positive affect and negative affect subscales were found to be equal to 0.88.

#### Perceived stress

The Italian translation of the Perceived Stress Scale (PSS; Cohen et al., 1983) by Andrea Fossati (2010) was used to measure teachers’ perception of stress. The PSS was developed on the basis of Lazarus’s theory of stress appraisal (Lazarus, 1999; Lazarus & Folkman, 1984). The PSS assesses the feelings and thought of people regarding stressful life situations. The PSS is composed of ten items that aim to measure the degree to which people experience their life as uncontrollable, unpredictable, and overloaded. The questions are about feelings and thoughts during the last month. The items are aimed to assess the current perceived stress levels. For each item, teachers were asked to indicate how often they felt a specific way on a 5-point Likert scale ranging from 0 (*never*) to 4 (*very often*). Higher scores indicate a higher level of perceived stress. Cronbach’s alpha was equal to 0.88.

#### Coping strategies

Coping Orientation to the Problems Experienced-New Italian Version-25 (COPE-NVI-25; Foà et al., 2015) was used to assess the coping strategies utilized by teachers. COPE-NVI-25 is the reduced version of COPE-NVI, derived from the COPE inventory by Carver and colleagues (Carver et al., 1989; Sica et al., 2008). This instrument is aimed at assessing people’s coping styles through five independent dimensions: problem orientation, positive attitude, avoidance, social support, and transcendent orientation. Problem orientation refers to use of planning and active coping strategies. Positive attitude involves acceptance and positive reinterpretation of negative events. Avoidance refers to use of strategies such as denial and behavioural and mental disengagement. Social support involves seeking of information, sympathy, or understanding from other people. Transcendent orientation is the tendency to turn to religion. COPE-NVI-25 consists of 25 items that can be answered via a 4-point Likert scale ranging from 1 (*I usually do not do this at all*) to 4 (*I almost always do this*). The items ask people to focus on what they usually do when facing a stressful experience. The authors reported acceptable values of Cronbach’s alpha (ranging from 0.63 to 0.96) and a validity equivalent to that of the original COPE. Cronbach’s alphas for social support, avoidance, positive attitude, problem orientation, and transcendent orientation scales were found to be equal to 0.80, 0.48, 0.74, 0.65, and 0.95 respectively.

### Data analysis

Statistical analyses were performed using the R software (version 1.4.1717). The “psych” package (version 2.1.9) and the “stats” package (version 4.1.2) were used to derive descriptive statistics and run regression analyses, respectively. Means and standard deviations, ranges, and percentages were computed to describe participants’ socio-demographics, characteristics of their teaching career, and experience with online teaching. The bivariate associations between variables of interest were assessed using Pearson’s correlation. Finally, three hierarchical linear regression analyses were run to evaluate the associations between participants’ emotion regulation, coping strategies, years of teaching experience, perceived workload, and attitudes towards online teaching introduced as predictors, and perceived stress, and positive and negative affect selected as outcomes of the study, after controlling for socio-demographic variables. In all the regressions, age, gender, and having children were entered into each model as step one; at step 2, years of teaching, perceived workload, factors related to online teaching experience, emotion regulation strategies (expressive suppression and cognitive reappraisal), and coping strategies (social support, avoidance, positive attitude, problem orientation, and transcendent orientation) were entered into the models. Assumptions for multiple regression were examined for the three regression models and no relevant violations were found.

## Results

Teachers reported a current significant increase of their workload (*M* = 5.96, *SD* = 1.05, range = 1–7). Table 2 shows teachers’ perceptions and attitudes towards online teaching. The teachers reported they had limited previous experience of online teaching (*M* = 2.87); however, they considered the resources available for online teaching as quite adequate (*M* = 6.05). The teachers perceived online teaching as a quite stressful experience (*M* = 7.28) and reported on average they were unwilling to continue working online (*M* = 7.31).

**Table 2:** Participants’ perceptions and attitudes towards teaching online

Variable	Gender		
	Female (*n* = 988)	Male (*n* = 190)	Total (*N* = 1178)
	*M* (*SD*)	*M* (*SD*)	*M* (*SD*)
Previous online teaching experience	2.87 (3.48)	3.25 (3.61)	2.93 (3.51)
Confidence in using educational technologies	6.62 (2.47)	7.52 (2.14)	6.77 (2.44)
Good quality of interactions with students	2.73 (2.56)	2.71 (2.69)	2.72 (2.58)
Perceived burden of online teaching	7.28 (2.74)	6.91 (2.87)	7.22 (2.77)
Adequacy of available resources for online teaching	6.05 (2.63)	6.19 (2.57)	6.07 (2.62)
Unwillingness to use online teaching	7.31 (3.06)	7.31 (3.23)	7.31 (3.08)

*M*, mean; *SD*, standard deviation

Table 3 indicates the means and standard deviations of the COPE-NVI-25, ERQ, PANAS, and PSS-10 measures, and correlations between them.

**Table 3:** Means, standard deviations of COPE-NVI-25, ERQ, PANAS, and PSS-10 and correlations between them

Variable	*M*	*SD*	1	2	3	4	5	6	7	8	9
1. Positive attitude	3.03	0.56									
2. Avoidance	1.35	0.34	− 0.13***								
3. Problem orientation	2.96	0.52	0.44***	− 0.18***							
4. Transcendent orientation	1.90	0.99	0.11***	0.05	0.08**						
5. Social support	2.51	0.65	0.12***	− 0.004	0.31***	0.15***					
6. Cognitive reappraisal	5.16	1.15	0.50***	− 0.08**	0.30***	0.12***	0.12***				
7. Expressive suppression	3.19	1.30	− 0.04	0.15***	0.03	0.04	− 0.26***	0.07*			
8. Positive affect	29.85	7.46	0.38***	− 0.18***	0.34***	0.04	0.08**	0.34***	− 0.02		
9. Negative affect	22.24	7.78	− 0.27***	0.26***	− 0.07*	0.05	0.15***	− 0.22***	− 0.00	− 0.35***	
10. Perceived stress	18.98	7.01	− 0.30***	0.23***	− 0.14***	− 0.04	0.12***	− 0.28***	− 0.01	− 0.52***	0.74***

*M*, mean; *SD*, standard deviation

^*^p < 0.05, **p < 0.01, ***p < 0.001

The results of the first hierarchical regression analysis predicting teachers’ perceived stress from the set of selected predictors are summarized in Table 4. In step 1, age, gender, and having children were entered into the model and only accounted for 4% of the variance in perceived stress scores after adjustment for the number of variables (adjusted *R*^2^ = 0.041, *F*(3, 1174) = 17.78, *p* < 0.001). Age and gender were significant predictors, with being younger and female predicting higher levels of perceived stress. In step 2, emotion regulation dimensions, coping strategies, years of teaching experience, perceived workload, and factors related to teachers’ perceptions and attitudes towards online teaching were entered. The model accounted for 27% of the variance in perceived stress scores adjusting for the number of variables (adjusted *R*^2^ = 0. 274, *F*(18, 1159) = 25.68, *p* < 0.001); the model showed a significant increase of about 23% in explained variance in perceived stress. With specific regard to emotion regulation, a small significant positive association was found between greater use of expressive suppression and a higher level of stress; on the other hand, a stronger attitude towards using cognitive reappraisal was associated with a lower level of perceived stress. With regard to coping strategies, a negative association with perceived stress was found for positive attitude; on the contrary, a higher tendency to use avoidance and higher reported social support were associated with greater stress. Perceived workload, confidence in using educational technologies, and burden of online teaching were all significant predictors of stress.

**Table 4:** Summary of hierarchical regression analysis for variables predicting teachers’ perceived stress

Predictor	Step			
	1		2	
	*B*	*β*	*B*	*β*
Age	− 0.11	− 0.18***	− 0.08	− 0.11*
Gender	2.76	0.14***	2.63	0.14***
Having children	− 0.14	− 0.01	− 0.07	− 0.01
Years of teaching experience			− 0.03	− 0.04
Perceived workload			1.48	0.22***
Online teaching experience			− 0.08	− 0.04
Confidence in using educational technologies			− 0.19	− 0.07*
Good quality of interactions with students			− 0.10	− 0.04
Burden of online teaching			0.22	0.09**
Adequacy of resources for online teaching			− 0.10	− 0.04
Unwillingness to use online teaching			− 0.11	− 0.05
Cognitive reappraisal			− 1.08	− 0.18***
Expressive suppression			0.32	0.06*
Positive attitude			− 2.21	− 0.18***
Avoidance			3.51	0.17***
Problem orientation			− 0.29	− 0.02
Transcendent orientation			− 0.29	− 0.04
Social support			1.46	0.13***
*R*^2^		0.043***		0.285***
Adjusted *R*^2^		0.041***		0.274***
Δ*R*^2^				0.233***

*B*, regression coefficient; *β*, standardized regression coefficient; *R*^2^, variance; adjusted *R*^2^, variance corrected for the number of predictors; Δ*R*^2^, change in *R*^2^

^*^p < 0.05, **p < 0.01, ***p < 0.001

Table 5 reports the results of the regression analysis of negative affect. Consistent with the results of the first regression, age and gender were significant predictors of negative affect in the first step model. The model accounted for about 6% of the variance in negative affect (adjusted *R*^2^ = 0.056, *F*(3, 1174) = 24.43, *p* < 0.001). In step 2, the other relevant predictors of negative affect were entered into the model. This model accounted for 24% of the variance in negative affect (adjusted *R*^2^ = 0.241, *F*(18, 1159) = 21.81, *p* < 0.001); use of cognitive reappraisal, positive attitude, avoidance, and perceived social support were significant predictors of levels of teachers’ negative affect in addition to age, gender, and perceived workload and burden of online teaching. In line with the results of the first regression, a more marked tendency to use cognitive reappraisal and having a positive attitude were associated with lower negative affect; on the other hand, use of avoidance and perceived social support were associated with a higher level of negative affect.

**Table 5:** Summary of hierarchical regression analysis for variables predicting teachers’ negative affect

Predictor	Step			
	1		2	
	*B*	*β*	*B*	*β*
Age	− 0.16	− 0.21***	− 0.12	− 0.15***
Gender	2.94	0.14***	2.75	0.13***
Having children	− 0.03	− 0.01	0.03	0.01
Years of teaching experience			− 0.03	− 0.04
Perceived workload			1.20	0.16***
Online teaching experience			− 0.06	− 0.03
Confidence in using educational technologies			− 0.09	− 0.03
Good quality of interactions with students			− 0.01	− 0.01
Burden of online teaching			0.29	0.10***
Adequacy of resources for online teaching			− 0.06	− 0.02
Unwillingness to use online teaching			− 0.12	− 0.05
Cognitive reappraisal			− 0.97	− 0.14***
Expressive suppression			0.25	0.04
Positive attitude			− 2.74	− 0.20***
Avoidance			4.82	0.21***
Problem orientation			0.83	0.06
Transcendent orientation			0.35	0.04
Social support			1.44	0.12***
*R*^2^		0.058***		0.253***
Adjusted *R*^2^		0.056***		0.241***
Δ*R*^2^				0.185***

*B*, = regression coefficient; *β*, standardized regression coefficient; *R*^2^, variance; adjusted *R*^2^, variance corrected for the number of predictors; Δ*R*^2^, change in *R*^2^

^*^p < 0.05, **p < 0.01, ***p < 0.001

Finally, Table 6 shows the results of the last regression analysis predicting positive affect from the selected variables. In the first step, the socio-demographic variables were entered and the model was not significant (adjusted *R*^2^ = 0.002, *F*(3, 1174) = 1.697, *p* = 0.166). In the last step, the relevant predictors were entered as in the previous models; the model accounted for 25% of the variance in positive affect (adjusted *R*^2^ = 0.252, *F*(18, 1159) = 23.02, *p* < 0.001). A stronger tendency towards using cognitive reappraisal was associated with higher levels of positive affect. Similarly, among coping strategies, positive attitude and problem orientation were associated with a more positive affective experience. Avoidance showed a negative association with a stronger disposition towards using this strategy being associated with lower levels of positive affect. Perceived workload, online teaching experience, and quality of interaction with students were all significant predictors of teachers’ positive affect.

**Table 6:** Summary of hierarchical regression analysis for variables predicting teachers’ positive affect

Predictor	Step			
	1		2	
	*B*	*β*	*B*	*β*
Age	− 0.01	− 0.01	0.02	0.03
Gender	− 0.56	− 0.03	− 0.84	− 0.04
Having children	1.01	0.06*	0.85	0.05
Years of teaching experience			− 0.04	− 0.05
Perceived workload			− 0.56	− 0.08**
Online teaching experience			0.13	0.06*
Confidence in using educational technologies			0.07	0.02
Good quality of interactions with students			0.31	0.11***
Burden of online teaching			− 0.03	− 0.01
Adequacy of resources for online teaching			0.16	0.05
Unwillingness to use online teaching			− 0.03	− 0.01
Cognitive reappraisal			1.15	0.18***
Expressive suppression			− 0.28	− 0.05
Positive attitude			2.53	0.19***
Avoidance			− 2.35	− 0.11***
Problem orientation			2.65	0.18***
Transcendent orientation			− 0.10	− 0.01
Social support			− 0.13	− 0.01
*R*^2^		0.004		0.263***
Adjusted *R*^2^		0.002		0.252***
Δ*R*^2^				0.250***

*B*, regression coefficient; *β*, standardized regression coefficient; *R*^2^, variance; adjusted *R*^2^, variance corrected for the number of predictors; Δ*R*^2^, change in *R*^2^

^*^p < 0.05, **p < 0.01, ***p < 0.001

## Discussion

The purpose of the present study was to contribute to the literature on investigation of predictors of perceived stress and affective experience among Italian teachers during the COVID-19 pandemic. Specifically, the study sought to evaluate the associations between emotion regulation and coping strategies, on the one hand, and teachers’ perceived stress and negative and positive affect respectively, on the other. As hypothesized, emotion regulation and coping strategies were significantly associated with all the selected measures of teachers’ perceived stress and affective experience.

The results suggest that cognitive reappraisal, expressive suppression, positive attitude, avoidance, and social support were significant predictors of perceived stress. As expected, a stronger tendency to use cognitive reappraisal was associated with a lower level of perceived stress; by contrast, a small significant positive association was found between greater use of expressive suppression and a higher level of perceived stress. These findings are in accordance with previous studies. For example, in a study conducted with Spanish teachers, it was found that emotion regulation ability was significantly and negatively associated with stress (Mérida-López et al., 2017). Moreover, expressive suppression was found to be positively associated with poorer perceived wellbeing (Gross, 2015; Gross & John, 2003; Hu et al., 2014). Consistent with other studies carried out during the COVID-19 pandemic, a higher tendency to use avoidance was associated with greater stress. In a study conducted among an international sample of language teachers during the COVID-19 conversion to online teaching, avoidance coping strategies were found to be significantly correlated with negative outcomes, such as stress, anger, and sadness (MacIntyre et al., 2020); additionally, the authors found that avoidance coping increased as stress increased. In another study conducted among the general Chinese population, avoidance coping strategies were found to be significantly correlated with higher perceived stress (Yu et al., 2020). A positive association between avoidance coping strategies and distress was found in a study carried out in the UK by Dawson and Golijani-Moghaddam (2020). Different from expectations based on previous studies, in the present study, higher reported social support was associated with greater stress. This result is not in line with the findings of the literature on coping strategies and stress that describe social support as a functional strategy to cope with stress (Carver et al., 1989). Moreover, social support has been identified as a protective factor against stress that can mitigate the potential negative effects of the pandemic (Gloster et al., 2020). In a study carried out during the COVID-19 pandemic, a similar finding was reported by Babore et al. (2020), who found that higher social support predicted higher distress among Italian healthcare professionals. As argued by Carver et al. (1989), seeking emotional social support is not always a useful strategy. The authors assert that during a stressful transaction, people can feel reassured by seeking social support, and using this strategy can facilitate utilization of problem-focused strategies. This coping strategy may not be very adaptive and stress may intensify when people focus on distress for a longer time, or if social support resources are only used to vent one’s feeling, instead of actively making an effort to deal with the stressor. A similar finding was observed by Sica et al. (2008), who found that social support was not associated with psychological wellbeing. Specifically, the authors observed a positive relationship between social support and measures of anxiety and depression. In this regard, Sica et al. (2008) too suggested that using social support and/or turning to religion may reinforce a passive attitude. According to the authors, this hypothesis would be corroborated by the fact that they found social support correlated with worry processes that are considered passive modalities to manage anxiety. Moreover, as coping strategies are situation-based (Carver et al., 1989), in specific stressful situations, such as COVID-19 pandemic, due to the restrictive measure, people that usually apply active coping strategies were possibly forced to use other typologies of coping strategies. So, in various situations, the efficacy of specific coping strategies can be different (Carver et al., 1989). This aspect should therefore be further investigated given the differences with the previous literature. Another aspect to be explored is the relationship between perceived stress and various typologies and forms of social support, such as subjective support, objective support, and the source of social support (for example family, colleagues, friends).

With regard to teachers’ affective experience, using cognitive reappraisal, positive attitude, avoidance, and perceived social support were found to be significant predictors of levels of negative affect. As expected, a more marked tendency to use cognitive reappraisal was associated with lower negative affect. This result is consistent with the literature on individual differences in the emotion regulation processes (Gross & John, 2003). Having a positive attitude was associated with lower negative affect. Using avoidance was associated with a higher level of negative affect. In a study carried out among a sample of adolescents, university students, and the general population by Ben-Zur (2009), it was found that problem-focused coping strategies were negatively related to negative affect, while avoidance coping strategies were found to be positively associated with negative affect. In a longitudinal study carried out by Zacher and Rudolph (2021) during the early stage of the COVID-19 pandemic in Germany, negative affect was found to be positively related to denial. Contrary to expectations, perceived social support was associated with a higher level of negative affect. This result is different from the finding by Zacher and Rudolph (2021), who found that negative affect was negatively related to using emotional support.

As expected from previous studies (Gross & John, 2003), a stronger tendency towards using cognitive reappraisal was associated with higher levels of positive affect. Among coping strategies, positive attitude and problem orientation were associated with a more positive affective experience. Avoidance showed a negative association with a stronger disposition towards using this strategy being associated with lower levels of positive affect. These results are in line with the findings by Ben-Zur (2009) who found that problem-focused coping strategies were positively associated with positive affect and avoidance coping strategies were found to be associated negatively with positive affect.

Regarding emotion regulation strategies, different from expectations based on the literature, expressive suppression was not a significant predictor of affective experience. Expressive suppression was expected to be positively associated with greater levels of negative affect and negatively associated with positive affect (Gross, 2015; Gross & John, 2003; Hu et al., 2014). However, as highlighted by two meta-analyses that examined the relationship between emotion regulation and some indicators of mental health, such as positive affect and negative affect, some studies found that this relationship is not always significant and other studies found inconsistent results (Fernandes & Tone, 2021; Hu et al., 2014). For instance, as expected, a study found that expressive suppression was negatively associated with positive affect, while it was not positively related to negative affect (Balzarotti et al., 2010). Moreover, different from expectations, in one study, expressive suppression correlated negatively with anxiety (Duan, 2005); similarly, a positive correlation between expressive suppression and life satisfaction was reported in another study (Hong, 2011). Expressive suppression is likely to be less effective in reducing the experience of negative emotions than cognitive reappraisal (Gross & John, 2003; Hu et al., 2014); moreover, some dimensions, such as the type of emotion (positive and negative) suppressed, may play a crucial role in the relationship between expressive suppression and positive affect (Fernandes & Tone, 2021). Additionally, restrictions due to COVID-19 may have limited the possibilities of using habitual emotion regulation strategies or forced people to use some strategies only partially; these aspects may have influenced the results of this study.

Perceived workload was a significant predictor of the selected outcomes in the three hierarchical linear regression analyses that were run for the purposes of the current study. Specifically, an increasing workload was associated with higher stress and with higher negative affect, while a lower level of perceived workload was associated with a higher positive affect. With regard to the predictive contribution of factors related to teachers’ experience of online teaching, an increasing perceived burden associated with online teaching was found to be positively related with both perceived stress and negative affect. Teachers with higher confidence in using educational technologies reported lower levels of perceived stress; moreover, the current results highlighted a positive association of reporting previous online teaching experience and adequate online interactions with students with positive affect. Similar to these findings, a study by Hidalgo-Andrade et al. (2021) found that teachers who had previous training and experience with online teaching reported lower levels of perceived stress; and a study by Kim et al. (2022) found that perceived workload contributed negatively to teachers’ mental health and wellbeing.

In line with previous studies (Flesia et al., 2020; Hidalgo-Andrade et al., 2021; Mazza et al., 2020), data analysis showed that age and gender were significant predictors of stress, with being younger and female predicting higher levels of perceived stress.

Finally, we have no adequate information on perceived stress and affective experience among teachers in the Italian teachers during the period before the COVID-19 outbreak to measure specific change during the pandemic. The present sample of teachers showed higher levels of perceived stress during COVID-19 epidemic than the general population in a non-emergency situation. Indeed, the mean of the PSS-10 in the present study was higher (*M* = 18.98; *SD* = 7.01) compared to the international normative values (*M* = 13.02; *SD* = 6.35) indicated by Cohen and Williamson (1988). Moreover, taking into consideration teachers’ affective experience, the mean of the positive affect was lower (*M* = 29.85; *SD* = 7.46) and the mean of the negative affect was higher (*M* = 22.24; *SD* = 7.78) compared to the international normative values indicated by Watson et al. (1988) relating to the affective experience felt by subjects during the past few weeks (positive affect, *M* = 32.0; *SD* = 7.0; negative affect, *M* = 19.5; *SD* = 7.0). These findings are in line with previous studies conducted during the COVID-19 pandemic (Flesia et al., 2020; Gloster et al., 2020).

## Limitations

This study has some limitations. First, the sample was not randomly selected, and using convenience sampling may limit the generalizability of the findings of this study. Indeed, teachers’ specific personality traits and motivational aspects may have influenced their decision to participate in the research study. Therefore, the data may not be representative of the entire teaching population. Second, due to the aim of this study, we only examined some variables as predictors of affective experience and perceived stress during COVID-19 pandemic. Future research is needed to explore other potential predictors of these variables. For instance, during the COVID-19 pandemic, specific personality traits, such as neuroticism, have been found to be related to distress (Margetić et al., 2021; Mazza et al., 2020), and psychological flexibility has been found to be positively related to positive affect and negatively related to negative affect (Gloster et al., 2020). Third, due to the cross-sectional nature of the study, no causal relationships between the measured variables can be inferred. It would be important to investigate whether a causal relationship exists. Fourth, the avoidance subscale of the COPE-NVI-25 showed a poor internal consistency in this study, and this may have affected the results to some extent; for example, this may have led to overestimation or underestimation of the correlation between the avoidance coping strategy and the other constructs. Finally, the data of this study were only collected during the COVID-19 pandemic, so we cannot measure any kind of change in perceived stress and affective experiences among teachers during the pandemic.

## Conclusions

This study contributes to research on investigation of predictors of perceived stress and affective experience among Italian teachers during the COVID-19 pandemic as they were forced to change their teaching methods and manage and regulate the emotions they experienced in response to the pandemic. As in previous studies, important findings on the role of coping and emotion regulation strategies on perceived stress and affective experience emerged from the data analyses. First, there is a positive association between using cognitive reappraisal and a lower level of perceived stress, and a positive association between using expressive suppression and a higher level of perceived stress; moreover, a more marked tendency to use avoidance-oriented coping strategies is associated with greater perceived stress. Second, a stronger tendency to use cognitive reappraisal and having a positive attitude are associated with lower negative affect; conversely, using avoidance coping strategies is associated with a higher level of negative affect. Third, a stronger tendency to use cognitive reappraisal, positive attitude, and problem orientation strategies is associated with a higher level of positive affect. To mitigate the psychological effect of the COVID-19 pandemic on teachers, the results of this study and similar previous studies could be useful for providing teacher training paths on emotion regulation and coping strategies to reduce stress and promote positive affect.

## Data Availability

The aggregate data are available from the corresponding author on reasonable request.
